# Comparative analysis of nnU-Net and Auto3Dseg for fat and fibroglandular tissue segmentation in MRI

**DOI:** 10.1117/1.JMI.12.2.024005

**Published:** 2025-04-16

**Authors:** Yasna Forghani, Rafaela Timóteo, Tiago Marques, Nuno Loução, Maria João Cardoso, Fátima Cardoso, Mario Figueiredo, Pedro Gouveia, João Santinha

**Affiliations:** a Champalimaud Foundation, Champalimaud Clinical Centre, Digital Surgery Lab, Lisboa, Portugal; b Faculdade de Medicina de Lisboa, Lisboa, Portugal; c Champalimaud Foundation, Champalimaud Clinical Centre, Breast Unit, Lisboa, Portugal; d Champalimaud Foundation, ABC Global Alliance, Lisboa, Portugal; e Universidade de Lisboa, Instituto Superior Técnico, Lisboa, Portugal

**Keywords:** nnU-Net, Auto3DSeg, fibroglandular tissue segmentation, breast MRI segmentation, medical image segmentation

## Abstract

**Purpose:**

Breast cancer, the most common cancer type among women worldwide, requires early detection and accurate diagnosis for improved treatment outcomes. Segmenting fat and fibroglandular tissue (FGT) in magnetic resonance imaging (MRI) is essential for creating volumetric models, enhancing surgical workflow, and improving clinical outcomes. Manual segmentation is time-consuming and subjective, prompting the development of automated deep-learning algorithms to perform this task. However, configuring these algorithms for 3D medical images is challenging due to variations in image features and preprocessing distortions. Automated machine learning (AutoML) frameworks automate model selection, hyperparameter tuning, and architecture optimization, offering a promising solution by reducing reliance on manual intervention and expert knowledge.

**Approach:**

We compare nnU-Net and Auto3Dseg, two AutoML frameworks, in segmenting fat and FGT on T1-weighted MRI images from the Duke breast MRI dataset (100 patients). We used threefold cross-validation, employing the Dice similarity coefficient (DSC) and Hausdorff distance (HD) metrics for evaluation. The F-test and Tukey honestly significant difference analysis were used to assess statistical differences across methods.

**Results:**

nnU-Net achieved DSC scores of 0.946±0.026 (fat) and 0.872±0.070 (FGT), whereas Auto3DSeg achieved 0.940±0.026 (fat) and 0.871±0.074 (FGT). Significant differences in fat HD (F=6.3020, p<0.001) originated from the full resolution and the 3D cascade U-Net. No evidence of significant differences was found in FGT HD or DSC metrics.

**Conclusions:**

Ensemble approaches of Auto3Dseg and nnU-Net demonstrated comparable performance in segmenting fat and FGT on breast MRI. The significant differences in fat HD underscore the importance of boundary-focused metrics in evaluating segmentation methods.

## 1 Introduction

Breast cancer remains a significant public health concern worldwide, with 2.26 million cases annually, representing 11.7% of total cancer diagnoses and impacting 24.5% of female cancer patients. In addition, breast cancer emerges as the leading cause of cancer-related deaths among women, contributing to 15.5% of annual female cancer deaths.^1^ Early detection and accurate diagnosis are critical in improving patient outcomes and reducing mortality rates. Magnetic resonance imaging (MRI) has emerged as a valuable tool in breast cancer screening and diagnosis due to its ability to provide volumetric high-resolution images with excellent contrast between fat and fibroglandular tissue (FGT).^2^ Fat and FGT segmentation play key roles in breast cancer diagnosis; for instance, the ratio of FGT area to breast volume, known as breast density, is a recognized risk factor for breast cancer.^2^^,^^3^ It also facilitates the creation of digital twins,^4^ which are virtual replicas of a patient’s anatomy and physiology, enabling personalized simulation, risk assessment, and treatment planning. These digital twins enhance surgical planning and, when combined with the integration of artificial intelligence (AI) and machine learning, pave the way for more precise and autonomous surgical interventions, further optimizing patient outcomes.^4^^,^^5^ Because manual segmentation is time-consuming, subjective, and prone to interobserver variability,^5^^,^^6^ in recent years, there has been a growing interest in developing automated algorithms to streamline FGT segmentation in MR images, mostly using deep learning (DL) methods. Recently, deep learning accomplished impressive results and several architectures have been proposed for medical image segmentation tasks.^6^^–^^10^ Another important aspect of automated segmentation is image preprocessing as medical image features, such as image size, voxel spacing, and class ratio, undergo significant variation.^11^ Moreover, medical images contain critical information that might be distorted by certain image transformations, such as cropping, padding, and resizing. Automated machine learning (AutoML) frameworks offer a potential solution to these challenges by automating the process of model selection, hyperparameter tuning, and architecture optimization, thereby reducing the reliance on manual intervention and expert knowledge.^11^^,^^12^ Among AutoML algorithms, nnU-Net and Auto3Dseg have emerged as prominent approaches, focusing specifically on biomedical image segmentation.

nnU-Net is an advanced DL segmentation method that adapts to various tasks in biomedicine by automatically configuring all essential steps, from preprocessing to postprocessing. This self-configuring model optimizes patch size and batch size based on image information, such as median shape and distribution of spacings, while also considering GPU limitations. nnU-Net uses different variations of the U-Net architecture, including 2D U-Net, 3D U-Net, and cascaded 3D U-Net.^11^ It has shown high performance in different segmentation applications, such as brain tumor segmentation,^13^ early ischemic change segmentation on noncontrast computed tomography (CT),^14^ lung lesion segmentation on CT images,^15^ and whole breast and FGT segmentation in dynamic contrast-enhanced (DCE) MRIs.^3^

Although nnU-Net focuses on U-Net architectures, Auto3DSeg uses non-U-Net networks and a recent variation of U-Net, a transformer-based architecture. Auto3DSeg is a state-of-the-art solution for 3D medical image segmentation. With minimal user input, it leverages MONAI and GPU technology to automate the segmentation process efficiently. In its default configuration, Auto3DSeg employs three primary 3D segmentation algorithms: Differentiable Network Topology Search (DiNTS),^16^ segmentation residual network (SegResNet),^17^ and Swin UNEt TRansformers (UNETR),^18^ each with its specialized training approach. SegResNet and DiNTS use convolutional neural network (CNN) designs, whereas Swin UNETR relies on a transformer-based architecture. Since 2022, approaches based on Auto3DSeg implementation have achieved the first rank in different segmentation challenges, such as head and neck tumor segmentation,^19^ ischemic stroke lesion segmentation,^20^ kidney and kidney tumor segmentation,^21^ and segmentation of the aorta.^22^

In this study, we conduct a comparative analysis of nnU-Net and Auto3Dseg for FGT segmentation in breast MRIs. Through a series of experiments and evaluations, we aim to assess the performance and efficiency of these methods in real-world clinical settings.

## 2 Materials and Methods

### 2.1 Dataset

**Fig. 1:**
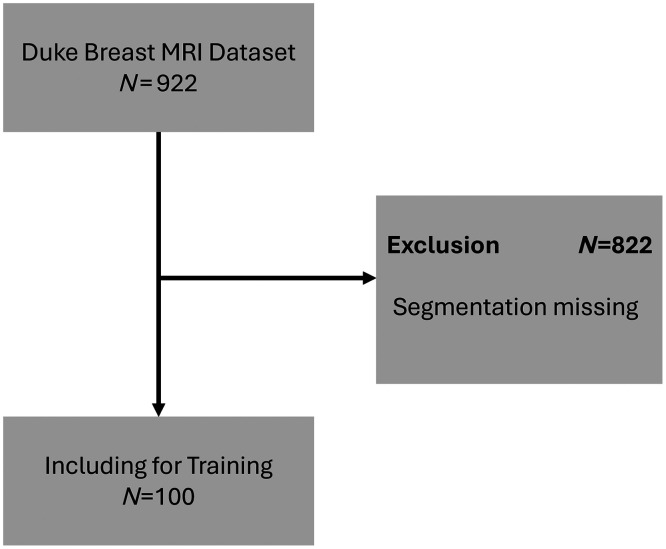
Summary of inclusion and exclusion criteria for the dataset.

In this study, we used the public Duke breast dataset, collected between 2000 and 2014 at the Duke Hospital, Durham, North Carolina, United States. This dataset contains axial breast MRIs of 922 patients, acquired with 1.5T or 3T scanners.^23^ The dataset contains breast tissue and FGT segmentations available in the initial pre-contrast phase of the DCE MRI sequences of 100 selected patients with segmented MRIs. The inclusion and exclusion criteria for the dataset are summarized in Fig. 1. We randomly split this dataset, with 75% used for training and the remainder for testing. Our approach involved threefold cross-validation to train and validate the segmentation methods. This proportion was selected to ensure sufficient data diversity during training while maintaining enough cases for robust testing. Despite the use of random splitting of the dataset, the different MRI manufacturers, molecular subtypes, and T-staging were represented in both training and test datasets, as shown in Appendix A, Table 4. The cases assigned to the training, testing, and each of the folds of cross-validation, provided in Appendix B (Table 5), were the same for both nnU-Net and Auto3Dseg to ensure a fair comparison. Dice similarity coefficient (DSC) and Hausdorff distance (HD) were used as evaluation metrics to validate the segmentation methods.

### 2.2 Segmentation Algorithms

Two AutoML segmentation tools, nnU-Net and Auto3DSeg, were employed to segment fat tissue and FGT in breast MRIs. These tools were selected for their demonstrated effectiveness in medical image segmentation and their ability to adapt to various datasets and tasks without requiring extensive manual adjustments. Both nnU-Net and Auto3DSeg comprise and test different architectures as well as their corresponding ensembles. An ensemble combines the outputs of multiple models to produce a final prediction, and in this study, majority voting was used to enhance robustness and accuracy.

#### 2.2.1 nnU-Net

nnU-Net framework automates the configuration and training of various U-Net models, including 2D U-Net, full-resolution 3D U-Net, and a 3D cascade. In the cascade setup, the first U-Net operates on downsampled images, whereas the second refines the segmentation maps generated at full resolution. nnU-Net introduces several architectural modifications to the original U-Net design, such as padded convolutions, instance normalization, and Leaky ReLUs, to enhance its performance and adaptability. Following cross-validation, nnU-Net autonomously selects the most effective configuration or ensemble based on empirical performance. The framework dynamically adjusts key hyperparameters, such as batch size, patch size, and pooling operations, to maximize spatial context while maintaining memory efficiency within GPU constraints. Learning rate scheduling ensures efficient convergence, with training stopped when the learning rate falls below a threshold or after a predefined number of epochs without significant performance change. In addition, nnU-Net integrates robust data augmentation techniques such as elastic deformations, random scaling, rotations, and gamma augmentation, crucial for enhancing model generalization.^11^

#### 2.2.2 Auto3DSeg

Auto3DSeg includes three different network architectures. The first is the DiNTS, which achieved very high scores in the Medical Segmentation Decathlon challenge.^16^ DiNTS introduces flexibility in topology design through a joint two-level search approach, ensuring optimal performance by minimizing the gap between continuous and discrete representations of network topologies. It incorporates a memory-aware search methodology, enabling the creation of 3D networks with varied GPU memory.^10^ The second architecture, SegResNet, employs an encoder–decoder CNN architecture. The encoder is asymmetrically larger to extract image information, whereas a smaller decoder is used to reconstruct the segmentation mask.^24^ The last architecture, the Swin UNETR neural network, combines the strengths of the Swin transformer and UNETR architectures. The Swin transformer, known for its efficient hierarchical self-attention mechanism using shifted windows, serves as the encoder in this U-shaped network. This design allows the encoder to extract features at multiple resolutions, leveraging the ability to capture detailed information across scales. These features are then connected to a CNN-based decoder via skip connections, enabling precise reconstruction of the input data.^18^ In an Auto3DSeg execution, each model has default training recipe transformers, which are presented in Table 1.

**Table 1:** Default training recipe transformers for each model.

Algorithm	DiNTS	SegResNet	Swin UNETR
Network	Densely connected lattice-based network	U-shape network architecture with 3D residual blocks	U-shape network architecture with Swin transformer–based encoder
Model input	96 × 96 × 96 (training and inference)	224 × 224 × 144 (training and inference)	96 × 96 × 96 (training and inference)
AMP	True	True	True
Optimizer	SGD	AdamW	AdamW
Initial learning rate	0.2	0.0002	0.0001
Loss	DiceFocalLoss	DiceLoss	DiceLoss
Transforms	-Intensity normalization	-Intensity normalization	-Intensity normalization
	-Random ROI cropping		
	-Random rotation	-Random ROI cropping	-Random ROI cropping
	-Random zoom	-Random affine transformation	-Random rotation
	-Random Gaussian smoothing	-Random Gaussian smoothing	-Random intensity shifting
	-Random intensity scaling	-Random intensity scaling	-Random flipping
	-Random intensity shifting	-Random intensity shifting	
		-Random Gaussian noising	
		-Random flipping	
	-Random Gaussian noising		

### 2.3 Segmentation Performance Evaluation

The DSC and HD metrics were used to assess the performance of the models on the cross-validation and held-out test for fat and FGT segmentation, with raincloud plots used to represent the DSCs and HDs obtained on the held-out test sets. Differences in test performances across all methods were assessed using the F-test with a significance level (α) of 5%,^25^ comparing individual algorithms and ensemble results. When statistical significance was observed, the post-hoc Tukey honestly significant difference (HSD) analysis was applied to identify the specific algorithms that exhibited statistically significant differences. This method inherently controls the family-wise error rate across the multiple pairwise comparisons conducted.

## 3 Results and Discussion

**Fig. 2:**
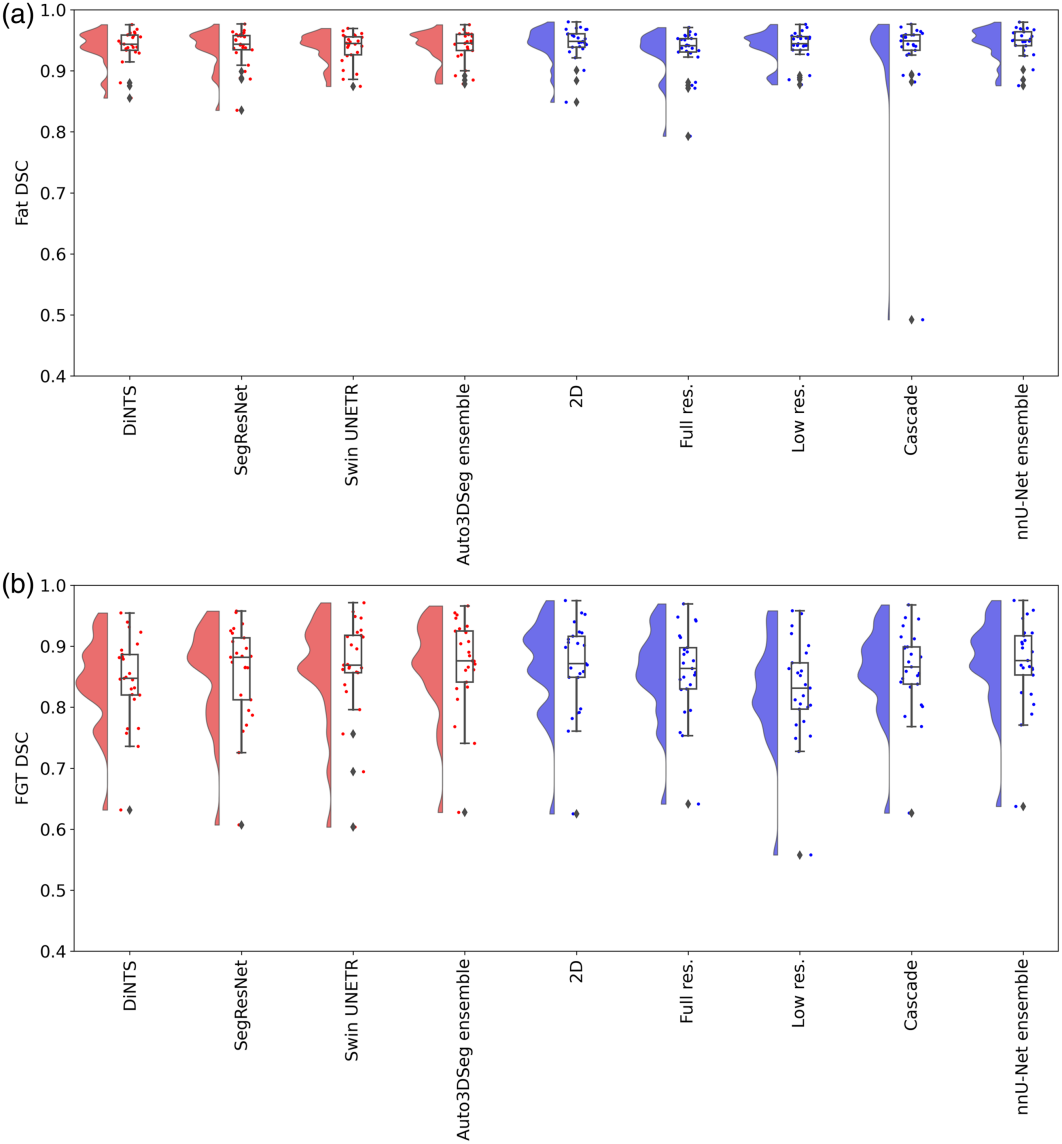
Raincloud plot of all algorithms for fat DSC (a) and FGT DSC (b).

**Fig. 3:**
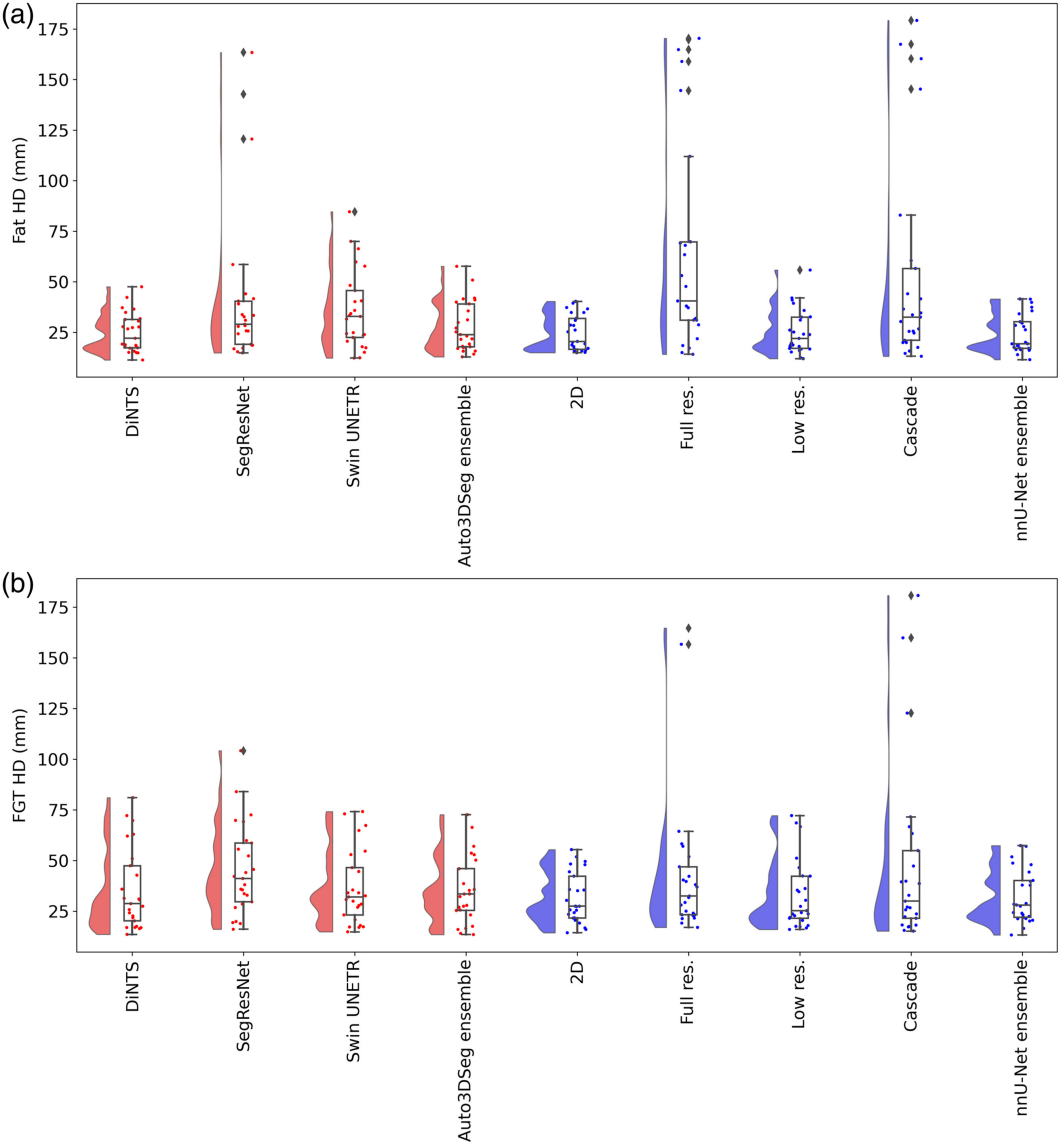
Raincloud plot of all algorithms for fat HD (a) and FGT HD (b).

Segmentations for 100 patients from the Breast Duke dataset^23^ were used (patient IDs are provided in Appendix B). The DSC and HD values (mean ± standard deviation, calculated across patients) for fat and FGT segmentation of each method for the test dataset are presented in Table 2, whereas the raincloud plots in Figs. 2 and 3 illustrate the distribution of DSC and HD for fat and FGT. As shown in Table 2, both nnU-Net and Auto3DSeg achieved high DSC scores across different network configurations, with ensemble methods performing better, even though not statistically significant. The nnU-Net ensemble achieved the highest fat DSC (0.946±0.026) and FGT DSC (0.872±0.070), closely followed by the Auto3DSeg ensemble, which achieved 0.940±0.026 and 0.871±0.074 for fat and FGT DSC, respectively. Although the observed differences in performance were not statistically significant, these findings suggest that ensemble models may enhance robustness and consistency in segmentation tasks by combining predictions from individual network configurations.

**Table 2:** Performance on the holdout test dataset for the segmentation of breast and FGT using nnU-Net and Auto3DSeg for different algorithms. Values are presented as mean ± standard deviation.

Method	Network algorithm	Fat DSC	FGT DSC	Fat HD (mm)	FGT HD (mm)
nnU-Net	2D	0.944 ± 0.029	0.867 ± 0.075	24.72 ± 8.77	**31.44 ± 12.33**
	3D low resolution	0.940 ± 0.026	0.828 ± 0.083	25.21 ± 10.95	32.85 ± 16.79
	3D full resolution	0.931 ± 0.038	0.858 ± 0.071	65.89 ± 53.69	44.59 ± 37.32
	3D cascade	0.926 ± 0.092	0.861± 0.069	53.16 ± 51.67	47.80 ± 44.17
	nnU-Net ensemble	**0.946 ± 0.026**	**0.872± 0.070**	**24.42 ± 9.40**	32.13 ± 13.05
Auto3DSeg	DiNTS	0.939 ± 0.029	0.844 ± 0.071	**24.85 ± 9.67**	35.43 ± 20.07
	SegResNet	0.937 ± 0.031	0.857 ± 0.080	42.48 ± 39.47	45.31 ± 22.09
	Swin UNETR	0.938 ± 0.025	0.866 ± 0.080	35.80 ± 19.37	36.67 ± 18.23
	Auto3DSeg ensemble	**0.940 ± 0.026**	**0.871 ± 0.074**	27.95 ± 12.32	**34.94 ± 16.24**

Note: bold values indicate the best performance for each metric (in each method): higher DSC and lower HD values represent superior segmentation accuracy and boundary delineation, respectively.

**Fig. 4:**
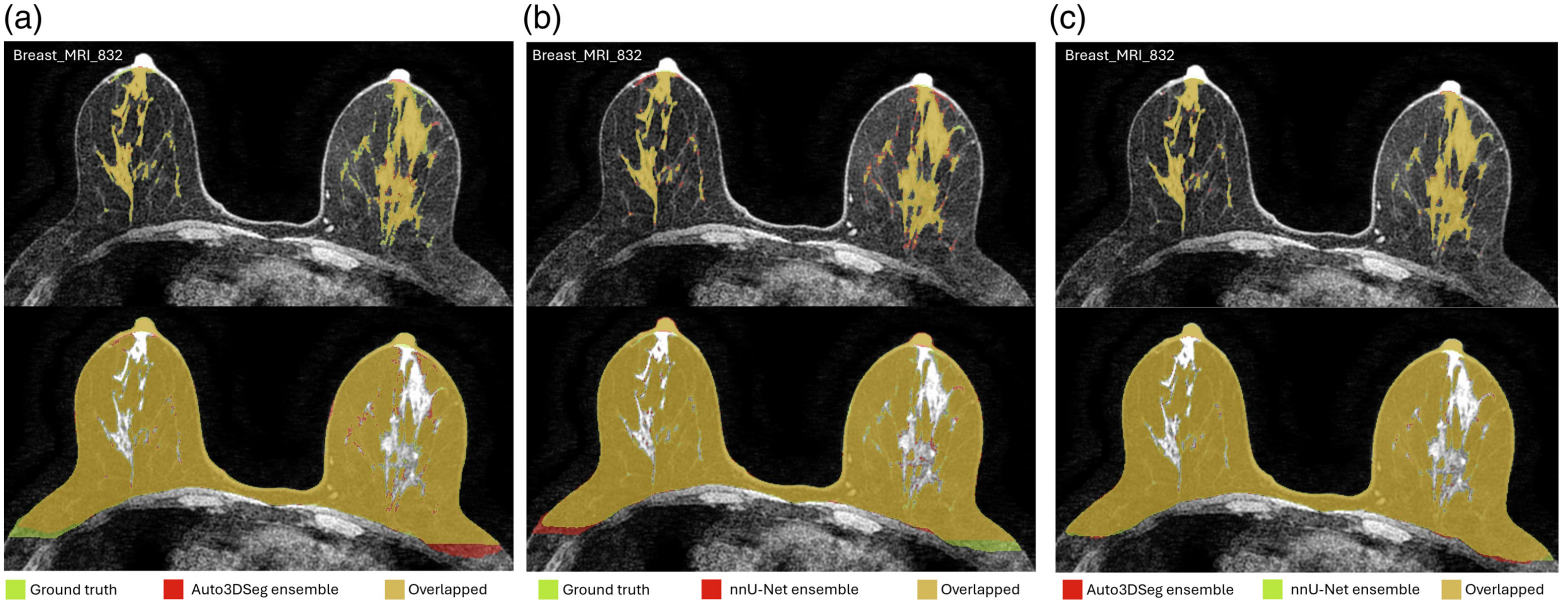
Representative case with the fat (lower row) and FGT (upper row) segmentations. (a) Ground truth and predicted labels by Auto3DSeg. (b) Ground truth and predicted labels by nnU-Net. (c) Predicted labels by Auto3DSeg and nnU-Net. The DSC values for Auto3DSeg were 0.945 (fat) and 0.871 (FGT) with HD values of 14.18 mm (fat) and 25.28 mm (FGT). For nnU-Net, the DSC values were 0.950 (fat) and 0.865 (FGT), with HD values of 13.93 mm (fat) and 28.38 mm (FGT).

**Fig. 5:**
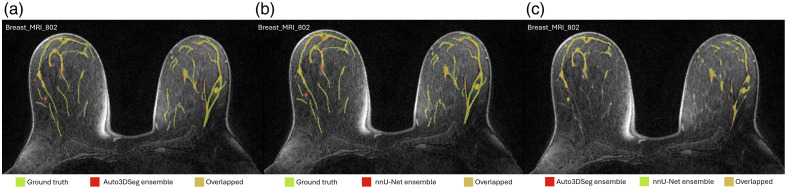
Example of a patient with low breast density causing a high standard deviation of FGT DSC. The DSC values for Auto3DSeg were 0.924 (fat) and 0.627 (FGT), with HD values of 27.10 mm (fat) and 52.81 mm (FGT). For nnU-Net, the DSC values were 0.926 (fat) and 0.637 (FGT), with HD values of 39.78 mm (fat) and 51.77 mm (FGT).

Figure 4 (zoomed-in view in Appendix C) illustrates a representative case of fat and FGT segmentation of Auto3DSeg and nnU-Net. Moreover, the performance plots for FGT DSC (Fig. 2) demonstrate a long tail toward lower values, resulting from the low density and thin properties of the FGT for a patient in the test set, as shown in Fig. 5 (zoomed-in view in Appendix C). In such scenarios, the lower number of voxels penalizes errors more strongly, a known shortcoming of DSC.^26^

We compared all methods together in the F-tests, including individual algorithms as well as ensemble results. In this study, no evidence for significant differences was observed for fat DSC (F=0.5424, p=0.8237), FGT DSC (F=0.8634, p=0.5483), or FGT HD (F=1.6270, p=0.1185). However, a significant difference was detected in fat HD (F=6.3020, p<0.001), indicating variability in boundary accuracy among configurations for fat segmentation. This difference can be explained by the characteristics of metrics and fat tissue. Fat tissue covers a large area, so small errors or shifts in pixel predictions have little effect on DSC, which measures how much the segmented volume overlaps with the true volume. However, HD is a boundary-focused metric and is more affected by such errors. Even slight misalignments along the edges can cause noticeable changes in HD values. This highlights the importance of paying attention to boundary accuracy when assessing segmentation performance for fat tissue.

Following the statistically significant differences across algorithms, determined through the F-test for the fat HD, the post-hoc Tukey HSD analysis revealed that full-resolution and 3D cascade U-Net were the primary contributors to the significant differences in fat HD, shown in Table 3 (with the complete comparison table provided in Appendix D). The full-resolution 3D U-Net is significantly different from all algorithms except SegResNet and cascade. In addition, cascade showed a statistically significant difference from the nnU-Net ensemble (p=0.022). These results emphasize the sensitivity of fat HD to specific network configurations.

**Table 3:** Pairwise comparisons of fat HD using Tukey HSD (significant differences only). The “p-adj” column represents the adjusted p-values obtained from the Tukey HSD test.

Group 1	Group 2	p-adj
Cascade	DiNTS	0.0259
Cascade	2D	0.0246
Cascade	Low res.	0.0296
Cascade	nnUnet ensemble	0.022
Full res.	DiNTS	0.0001
Full res.	Swin UNETR	0.0131
Full res.	Auto3DSeg ensemble	0.0004
Full res.	2D	0.0001
Full res.	Low res.	0.0001
Full res.	nnUnet ensemble	0.0001

The observed higher fat HD values may be explained by inconsistencies in defining the posterior limit of the breast fat. Specifically, some segmentation protocols exclude posterior tissues from the fat region, whereas neural networks may include these areas, leading to boundary disagreements. Unlike FGT, where boundaries are well-defined and circumscribed by fat, the ambiguity in fat segmentation boundaries likely contributes to the observed variability in fat HD.

This study aimed to compare the performance of nnU-Net and Auto3DSeg, two AutoML frameworks, in segmenting fat and FGT on breast MRI. Comparing individual methods across nnU-Net and Auto3DSeg, notable differences emerge, particularly in fat HD performance, where the cascade and full-resolution methods underperform due to higher HD values and variability (an example is shown in Appendix C), highlighting their limitations in accurately capturing boundary details in structures with hill defined limits. The cascade method exhibited a unique outlier in fat DSC, where voxels in the background region outside the patient were misclassified as fat in one case, a behavior not observed in other algorithms or cases (shown in Appendix C). This suggests an architectural limitation in the cascade method, possibly derived from its integration of low- and high-resolution predictions. Furthermore, the low-resolution approach struggles with small structures such as FGT, failing to recover fine details critical for accurate segmentation, as reflected in its lower FGT DSC values. DiNTS also performed well, particularly in fat HD, benefiting from its topology optimization capabilities.

We analyzed the performance of various algorithms across different molecular subtypes and tumor staging (T-staging) for fat and FGT segmentation, shown in Figs. 10 and 11 of Appendix E. The analysis shows that fat DSC is lower for patients with triple-negative and T3-stage tumors, indicating challenges in segmenting more aggressive breast cancer types. Although no clear HD trend is observed for molecular subtypes, models such as DiNTS, 2D U-Net, Low-Res. U-Net, and nnU-Net ensembles achieve lower HD. For FGT, DSC is highest for triple-negative patients, and T3-stage tumors generally have higher DSC across models except for SegRes. HD is lower for ER/PR-positive and HER2-positive patients but larger for T3-stage tumors. However, statistical significance was not assessed due to sample size limitations.

Both nnU-Net and Auto3DSeg ensembles failed to show any statistically significant differences in terms of DSC and HD, indicating that the ensemble approaches in both frameworks are equally effective for segmenting fat and FGT. However, in terms of computational cost (see Appendix F), Auto3DSeg methods (DiNTS, SegResNet, and Swin UNETR) generally converge later, resulting in significantly longer training times compared with other methods. For instance, DiNTS takes nearly 80 h, whereas SegResNet and Swin UNETR require 20.4 and 40.1 h, respectively—considerably more than nnU-Net methods, which range from 2.5 to 7.1 h. Although the segmentation accuracy achieved by these methods in this study is not statistically different, their extended training times and increased computational demands should be considered when selecting an appropriate method. The trade-off is that these methods may offer advantages in terms of model complexity and flexibility, but their higher resource requirements may limit their practicality for certain applications.

Our results indicate that nnU-Net and Auto3DSeg demonstrate comparable performance in segmenting fat and FGT in breast MRI, with ensemble approaches. However, significant differences in fat HD highlight the sensitivity of boundary-focused metrics to network configurations, particularly for tissues with less clearly defined boundaries. These findings, combined with the observed impact of anatomical variability and density differences on DSC, emphasize the need to consider both voxel density and boundary accuracy when evaluating segmentation methods. Furthermore, the results suggest that future improvements in segmentation accuracy might be achieved through refined preprocessing techniques that better account for anatomical and imaging variability, rather than focusing solely on network architecture.

Although many studies on FGT segmentation in breast MRI exist, the diversity in datasets, image modalities, and methodologies makes direct comparisons of results challenging. Each study often uses different imaging protocols and preprocessing techniques, leading to variability in reported DSC. For instance, one study achieved a high DSC of 0.951 for FGT segmentation using a 2D U-Net with T1-weighted images without fat suppression.^27^ Similarly, there are reported FGT DSCs of 0.909 and 0.916 for nnU-Net and a transformer-based neural network, respectively, but using a combination of T1- and T2-weighted sequences.^28^ By contrast, a study using nnU-Net in dynamic contrast-enhanced MRIs, which normalized the intensity scale to [0, 255], achieved DSCs of 0.968 for whole breast and 0.877 for FGT.^3^ Meanwhile, a generative adversarial network approach for FGT segmentation reported a DSC of 0.87.^2^

Hu et al.^29^ on fully automated DL methods for FGT segmentation used the Duke dataset and fat-saturated gradient echo T1-weighted pre-contrast images, the same ones used in our study. That study, which also used a U-Net architecture, achieved DSC values of 0.879 for breast and 0.730 for FGT. Although Hu et al. resized images to 512×512 and normalized image intensities using 5th to 95th percentile normalization, nnU-Net started by harmonizing voxel spacing, performing matrix size-based patch selection, and applying z-score intensity normalization. The difference in performance for the nnU-Net results reported here underscores the importance of preprocessing and highlights the need to maintain image information through appropriate preprocessing, as this can significantly impact segmentation outcomes. This study has several limitations. The dataset used in this study may not capture the full variability observed in breast MRI datasets with different imaging protocols and patient demographics, potentially limiting the generalizability of the findings across diverse clinical settings. Incorporating multiple datasets from various sources could provide a more comprehensive assessment of the methods’ performance.

## 4 Conclusion

This study compared the performance of nnU-Net and Auto3DSeg in segmenting fat and fibroglandular tissue (FGT) on breast MRI, finding no evidence of statistical differences between the ensemble approach of both frameworks. However, significant differences were observed in fat HD, reflecting the sensitivity of boundary-focused metrics to segmentation inconsistencies, especially for tissues with poorly defined boundaries. These results underscore the importance of considering both voxel-based and boundary-focused metrics when evaluating segmentation methods. Although Auto3DSeg methods required longer training times, they offered model flexibility and comparable segmentation accuracy. Future work should focus on refining preprocessing, incorporating diverse datasets, and addressing computational efficiency to enhance clinical applicability and generalization.

## 5 Appendix A: Summary Statistics of Dataset Variables

This appendix provides the proportion of cases by MRI manufacturers, molecular subtype, and tumor staging in the training and test datasets, allowing for a comparison of their distributions. This helps assess the representativeness of the datasets and ensures consistency in model evaluation.

**Table 4:** Proportion of cases by MRI manufacturer, molecular subtype, and tumor staging in the training and test datasets.

Variable		Train	Test
MRI manufacturer	Siemens	0.28	0.36
	GE	0.72	0.64
Molecular subtype	Luminal-like	0.67	0.6
	Triple-negative	0.08	0.12
	ER/PR pos, HER2 pos	0.04	0.04
	HER2	0.21	0.24
T-staging	1	0.42	0.64
	2	0.45	0.28
	3	0.09	0.08
	4	0.04	0

## 6 Appendix B: Patient IDs from the Duke Dataset

This appendix lists the patient IDs from the Duke dataset used in this study, organized by their respective training folds and test sets. These IDs represent the breast MRI scans included in the analysis.

**Table 5:** Patient IDs from the Duke dataset used in our study.

Test	Breast_MRI_686	Breast_MRI_025	Breast_MRI_681	Breast_MRI_080	Breast_MRI_705
	Breast_MRI_166	Breast_MRI_370	Breast_MRI_723	Breast_MRI_802	Breast_MRI_359
	Breast_MRI_726	Breast_MRI_501	Breast_MRI_503	Breast_MRI_495	Breast_MRI_279
	Breast_MRI_246	Breast_MRI_489	Breast_MRI_186	Breast_MRI_369	Breast_MRI_832
	Breast_MRI_741	Breast_MRI_670	Breast_MRI_286	Breast_MRI_124	Breast_MRI_797
Training fold 0	Breast_MRI_041	Breast_MRI_529	Breast_MRI_290	Breast_MRI_572	Breast_MRI_334
	Breast_MRI_329	Breast_MRI_392	Breast_MRI_229	Breast_MRI_105	Breast_MRI_595
	Breast_MRI_652	Breast_MRI_112	Breast_MRI_805	Breast_MRI_575	Breast_MRI_170
	Breast_MRI_636	Breast_MRI_089	Breast_MRI_525	Breast_MRI_773	Breast_MRI_054
	Breast_MRI_530	Breast_MRI_902	Breast_MRI_383	Breast_MRI_435	Breast_MRI_561
Training fold 1	Breast_MRI_302	Breast_MRI_238	Breast_MRI_602	Breast_MRI_888	Breast_MRI_587
	Breast_MRI_819	Breast_MRI_354	Breast_MRI_781	Breast_MRI_031	Breast_MRI_018
	Breast_MRI_788	Breast_MRI_230	Breast_MRI_616	Breast_MRI_339	Breast_MRI_492
	Breast_MRI_337	Breast_MRI_006	Breast_MRI_762	Breast_MRI_002	Breast_MRI_148
	Breast_MRI_440	Breast_MRI_497	Breast_MRI_143	Breast_MRI_876	Breast_MRI_363
Training fold 2	Breast_MRI_618	Breast_MRI_426	Breast_MRI_021	Breast_MRI_204	Breast_MRI_640
	Breast_MRI_280	Breast_MRI_612	Breast_MRI_338	Breast_MRI_688	Breast_MRI_287
	Breast_MRI_693	Breast_MRI_320	Breast_MRI_694	Breast_MRI_141	Breast_MRI_464
	Breast_MRI_562	Breast_MRI_023	Breast_MRI_466	Breast_MRI_272	Breast_MRI_528
	Breast_MRI_727	Breast_MRI_553	Breast_MRI_076	Breast_MRI_827	Breast_MRI_087

## 7 Appendix C: Supplemental Images

**Fig. 6:**
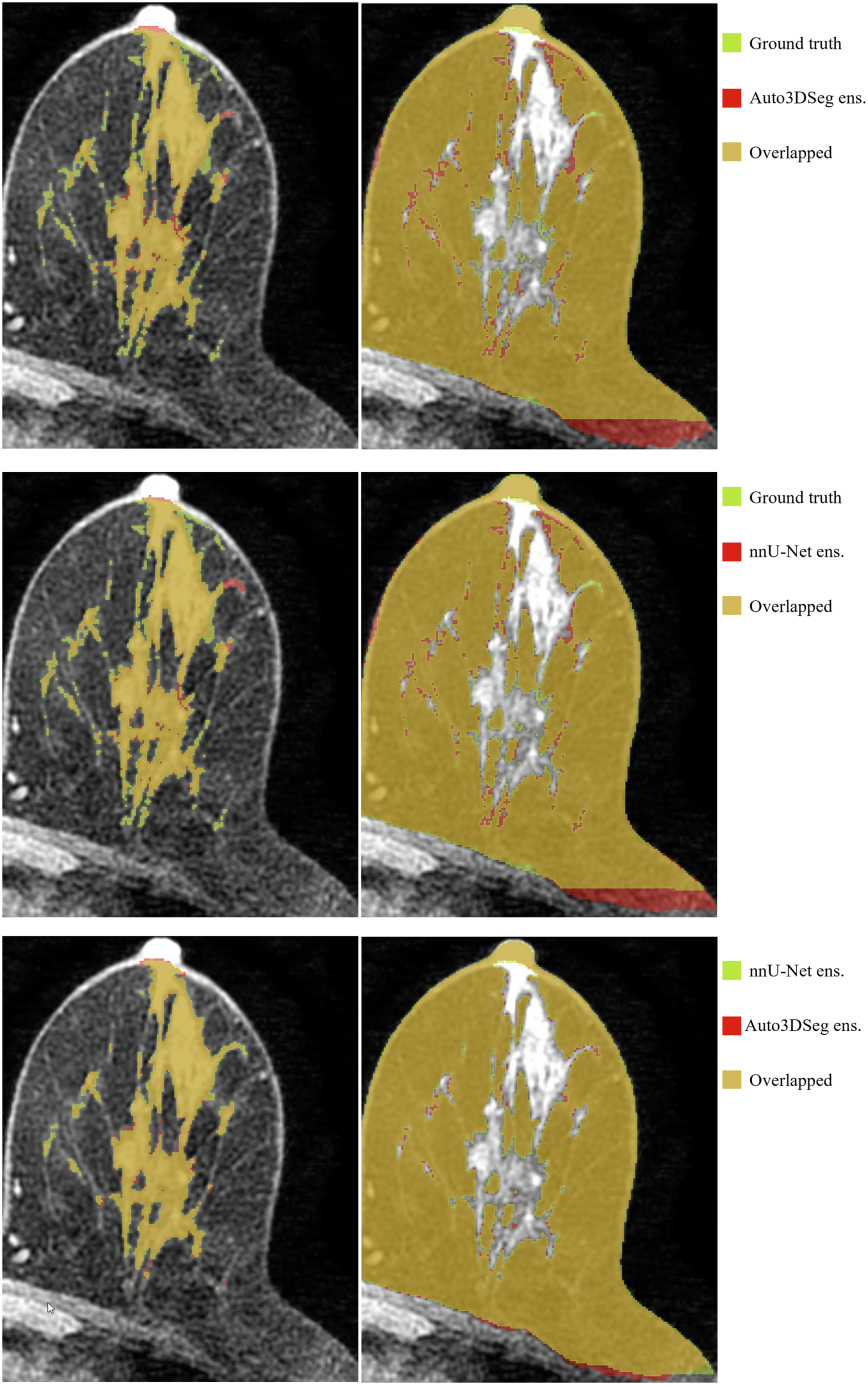
Zoomed-in view of Fig. 4.

**Fig. 7:**
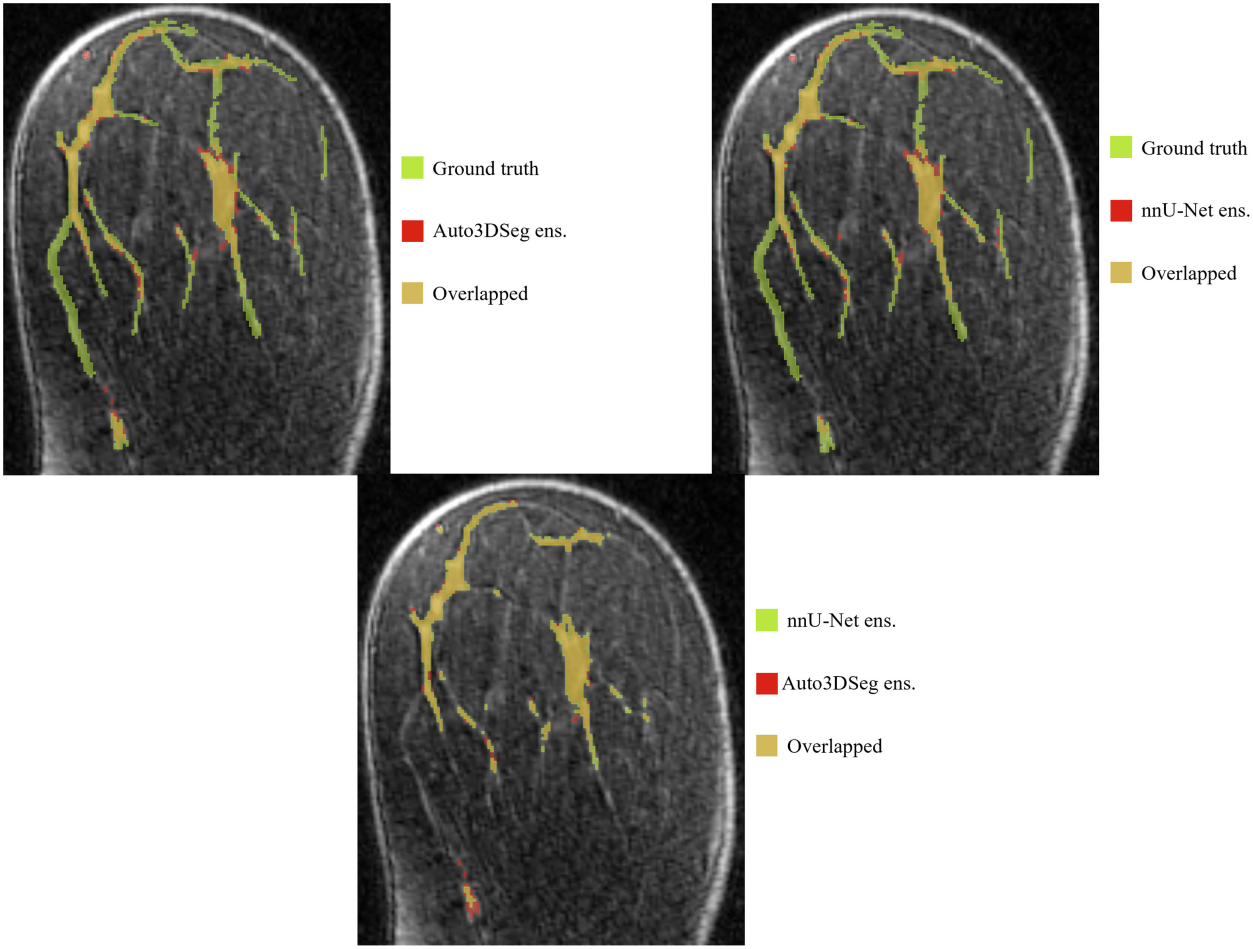
Zoomed-in view of Fig. 5.

**Fig. 8:**
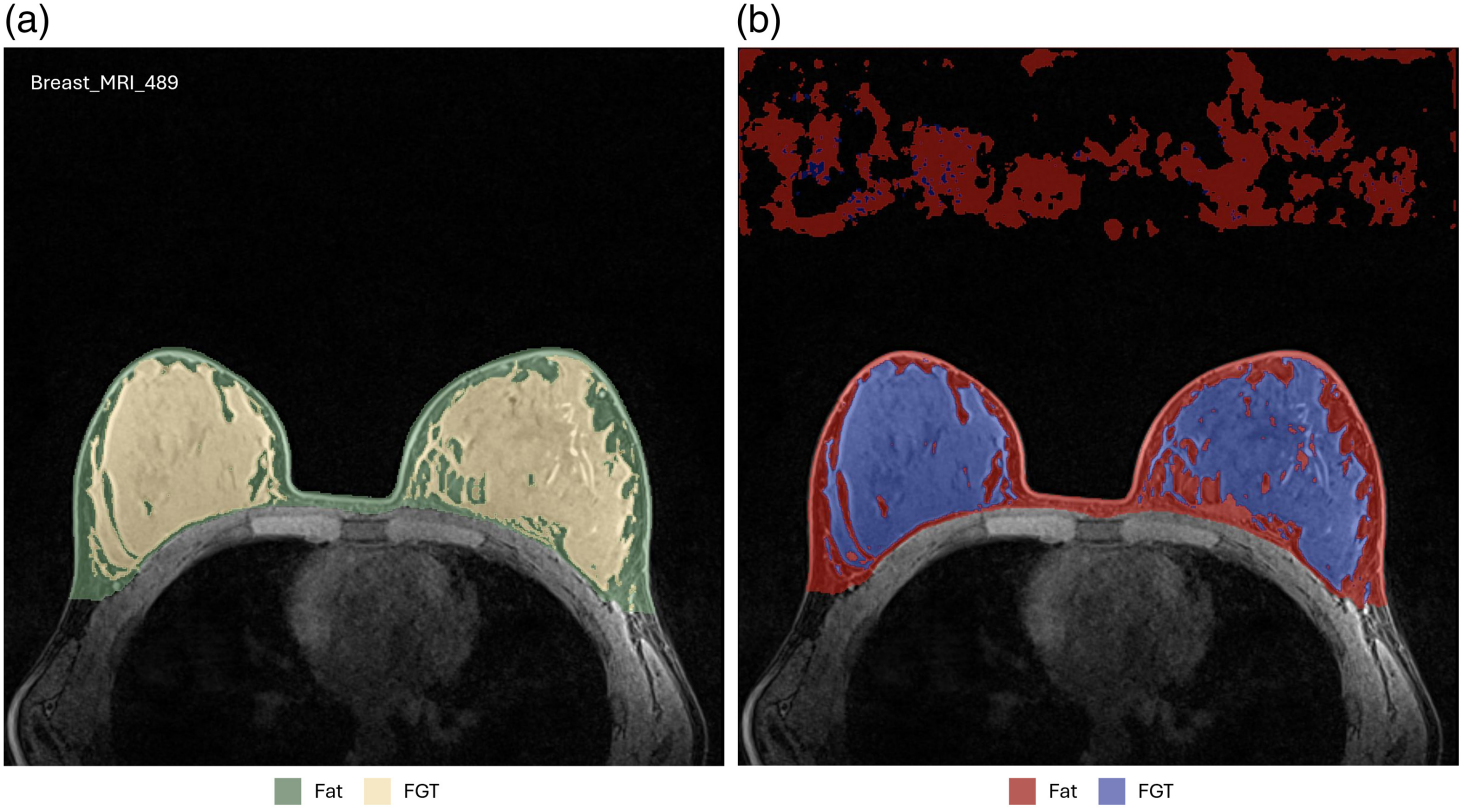
Unique outlier in fat DSC with cascade method (a) versus ground truth (b). The DSC for fat segmentation using the cascade method was 0.492, with an HD of 179.20 mm, compared with the ground truth HD of 180.72 mm.

**Fig. 9:**
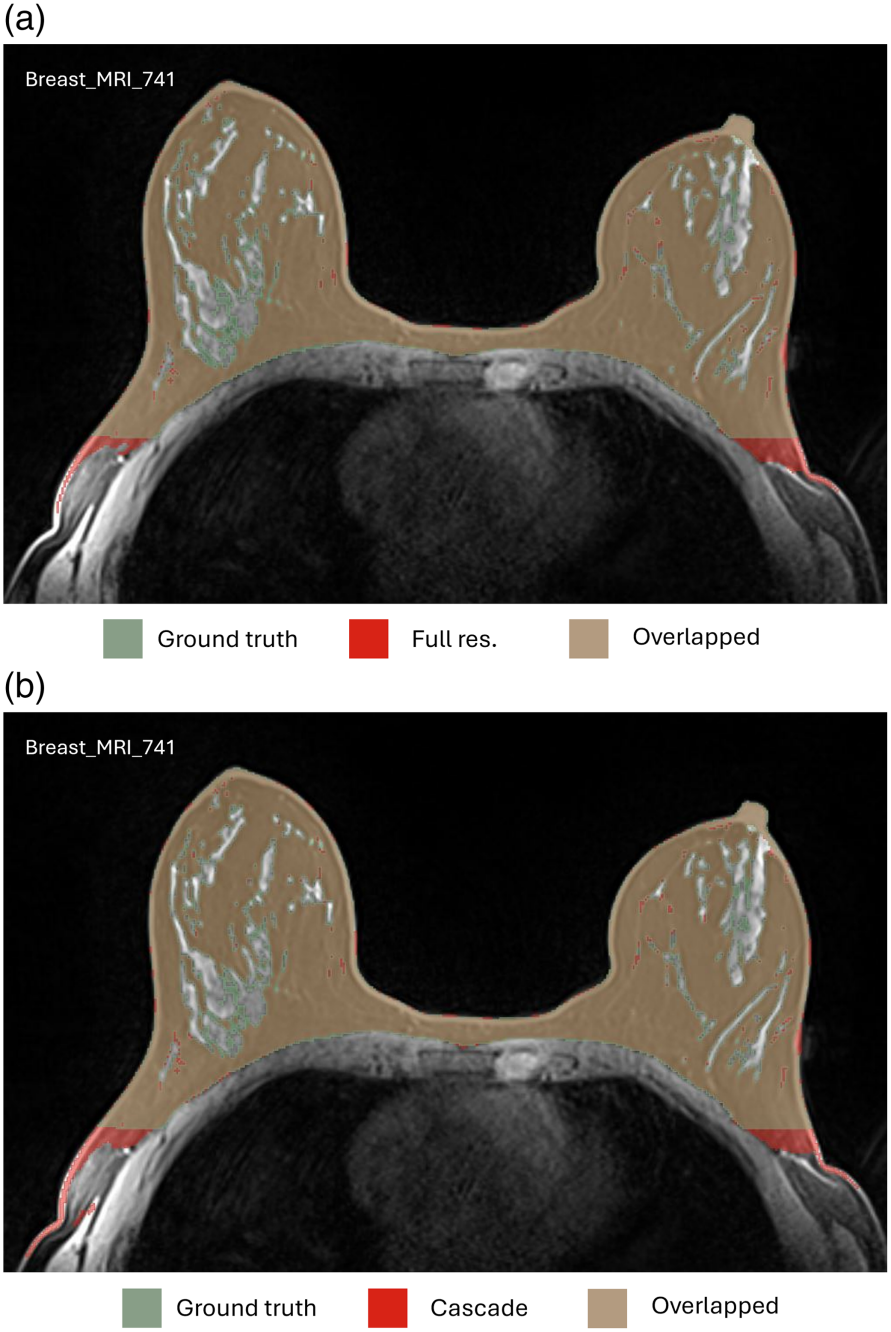
Example MRI showing fat segmentation with high HD in the full resolution model (a) and cascade model (b), compared with ground truth. The DSC values for the full resolution were 0.937 (fat) and 0.845 (FGT) with HD values of 164.77 mm (fat) and 164.65 mm (FGT). For cascade, the DSC values were 0.926 (fat) and 0.866 (FGT), with HD values of 167.41 mm (fat) and 26.66 mm (FGT).

This appendix provides additional visual examples and detailed insights into the studys findings. Figures 6 and 7 present zoomed-in views of Figs. 4 and 5, respectively, to highlight finer details. Figure 8 illustrates a unique outlier in the cascade method for fat DSC, where background tissue was misclassified as fat, revealing an architectural limitation. Figure 9 demonstrates an example of MRI comparing fat segmentation performance between the cascade and full-resolution models, emphasizing challenges in accurately capturing boundary details.

## 8 Appendix D: Fat HD Post-Hoc Comparison Table

This appendix provides the complete results of the post-hoc Tukey HSD analysis for fat HD. Table 6 displays all pairwise comparisons between methods, highlighting the statistically significant differences that contribute to the overall findings discussed in the paper.

**Table 6:** All pairwise comparisons of fat HD using Tukey HSD.

Group 1	Group 2	p-adj
Cascade	DiNTS	0.0259
Cascade	SegResNet	0.9402
Cascade	Swin UNETR	0.5059
Cascade	Auto3DSeg ensemble	0.0753
Cascade	2D	0.0246
Cascade	Full res.	0.8511
Cascade	Low res.	0.0296
Cascade	nnUnet ensemble	0.022
Full res.	DiNTS	0.0001
Full res.	SegResNet	0.1297
Full res.	Swin UNETR	0.0131
Full res.	Auto3DSeg ensemble	0.0004
Full res.	2D	0.0001
Full res.	Low res.	0.0001
Full res.	nnUnet ensemble	0.0001
Auto3DSeg ensemble	DiNTS	1
Auto3DSeg ensemble	SegResNet	0.733
Auto3DSeg ensemble	Swin UNETR	0.991
Auto3DSeg ensemble	2D	1
Auto3DSeg ensemble	Low res.	1
Auto3DSeg ensemble	nnUnet ensemble	1
nnUnet ensemble	DiNTS	1
nnUnet ensemble	SegResNet	0.4493
nnUnet ensemble	Swin UNETR	0.9153
nnUnet ensemble	2D	1
nnUnet ensemble	Low res.	1
2D	DiNTS	1
2D	SegResNet	0.733
2D	Swin UNETR	0.991
2D	Low res.	1
DiNTS	SegResNet	0.4842
DiNTS	Swin UNETR	0.9314
DiNTS	Low res.	1
Low res.	SegResNet	0.5138
Low res.	Swin UNETR	0.9432
SegResNet	Swin UNETR	0.997

## 9 Appendix E: Results by Subgroup

Appendix F presents the results of various algorithms evaluated across different molecular subtypes and tumor staging (T-staging) for fat and FGT segmentation. These results highlight patterns and tendencies observed in segmentation accuracy (DSC) and boundary agreement (HD) for different subgroup classifications.

**Fig. 10:**
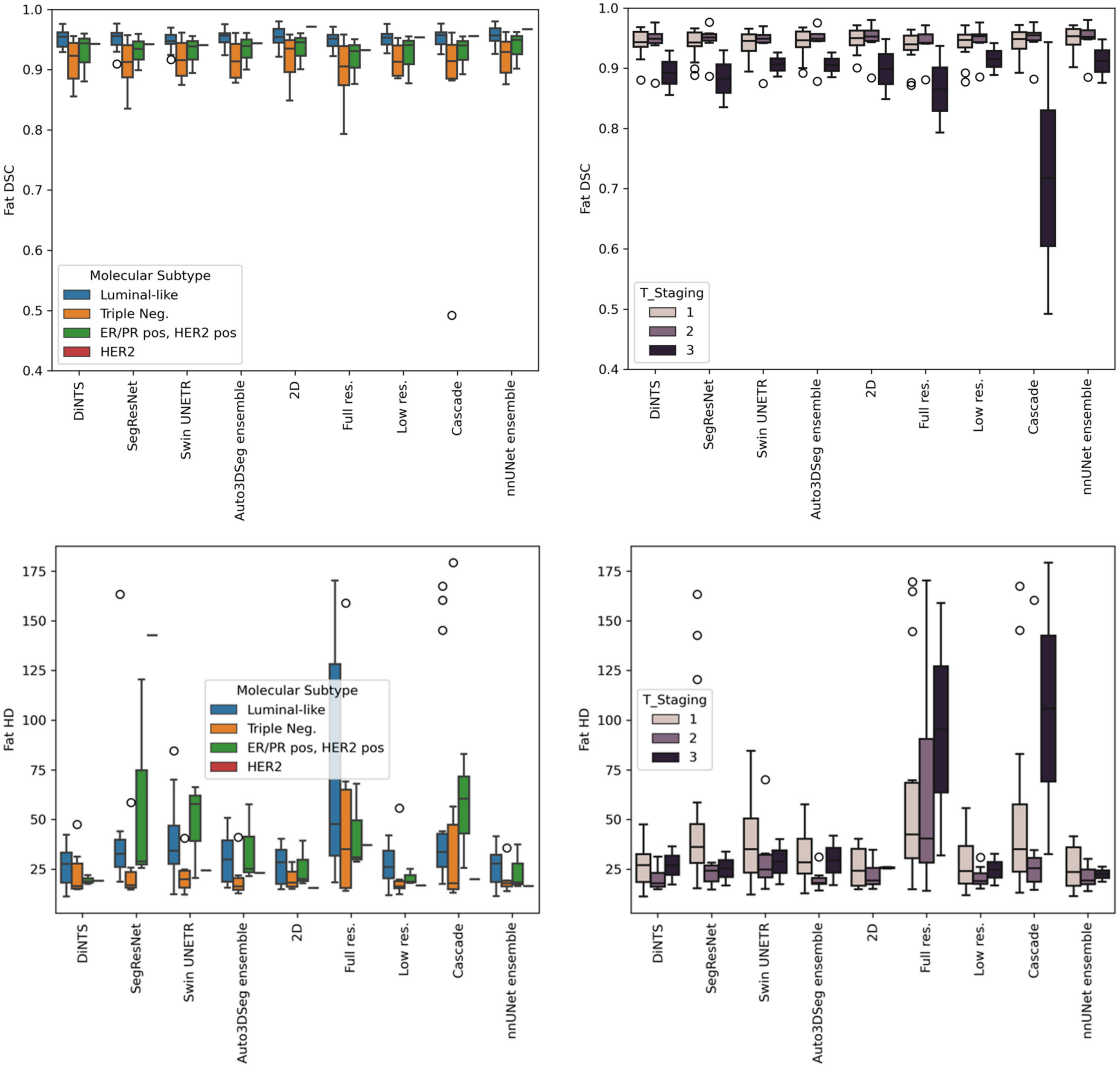
Fat DSC and HD results of all algorithms across molecular subtypes and T-staging.

Based on Fig. 10, it is possible to observe a tendency for a lower fat DSC in all models for the patients with a triple-negative molecular subtype and T3-staging, which reflect more aggressive types of breast cancer. When considering HD, no clear tendency is observed for a specific molecular subtype, but DiNTS, 2D U-Net, Low-Res. U-Net, and nnU-Net ensembles produce segmentations with lower HD across molecular subtypes. When considering the T-staging, we observe lower HD across stages in the nnU-Net ensemble, Low-Res. U-Net, 2D U-Net, DiNTS, SegRes, and Auto3DSeg ensemble.

**Fig. 11:**
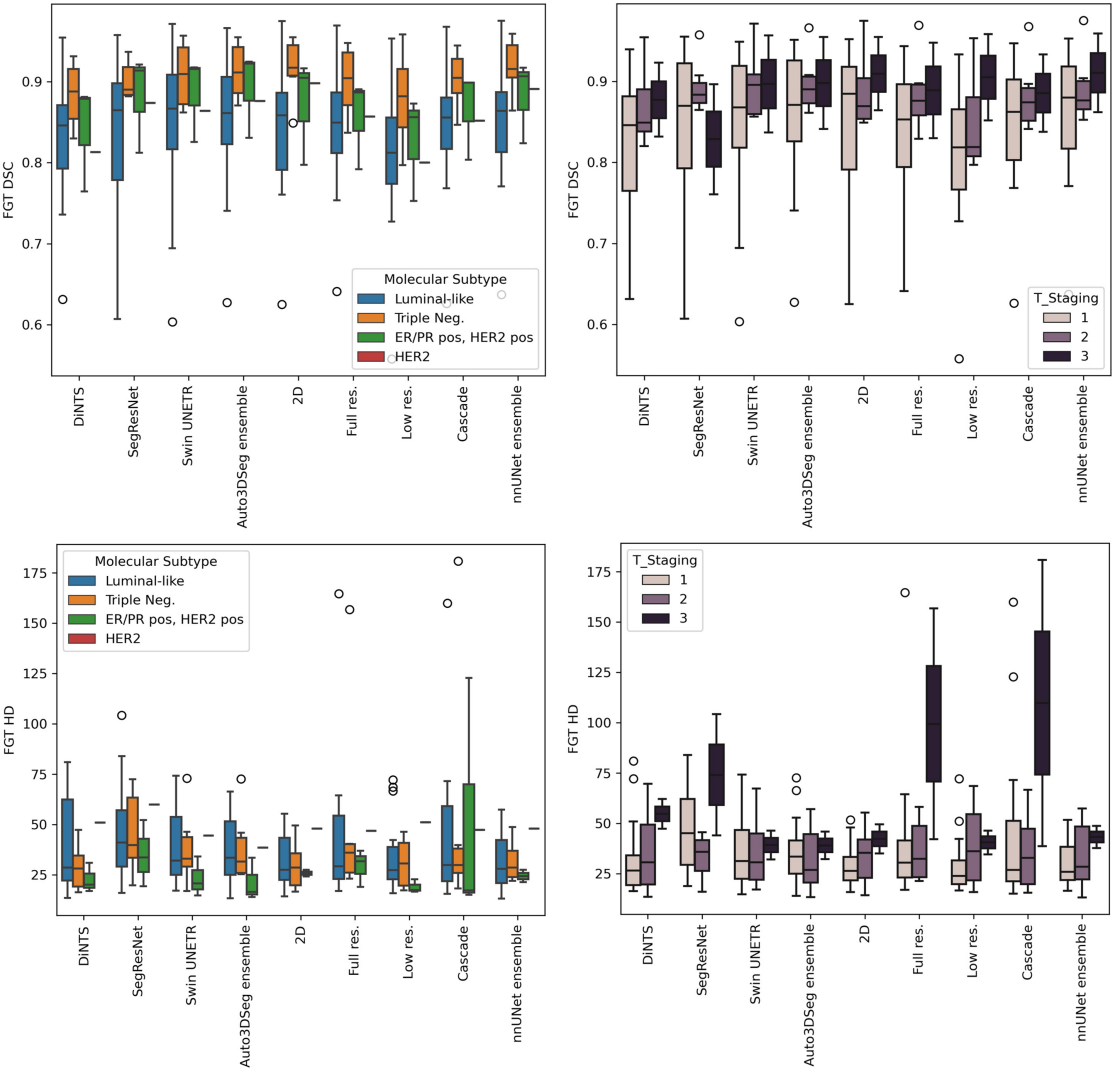
FGT DSC and HD results of all algorithms across molecular subtypes and T-staging.

Regarding the comparison of DSC and HD for the FGT, from Fig. 11, we observe an apparent higher DSC for patients with triple-negative molecular subtype, followed by ER/PR positive, HER2 positive, and Luminal-like. Considering T-staging, all algorithms except SegRes showed slightly higher DSC for T3, followed by T2 and T1. In terms of HD, all models presented lower HD for patients with ER/PR positive, HER2 positive molecular subtypes, where the cascade algorithm shows a larger interquartile range compared with other algorithms. Regarding T-staging, patients with T3-stage tumors showed larger HD.

Due to the test size and the number of groups, the differences between the molecular subtypes or the T-staging shown in Figs. 10 and 11 were not statistically assessed.

## 10 Appendix F: Training Time Comparison

This appendix provides a detailed comparison of training times and the number of epochs required for each method. Table 7 highlights the significantly longer training times of Auto3DSeg methods (DiNTS, SegResNet, and Swin UNETR) compared with other approaches, such as 2D, full-resolution, low-resolution, and cascade methods. These differences underscore the higher computational cost associated with Auto3DSeg methods, despite their performance advantages. All experiments were conducted using an NVIDIA RTX A6000 GPU, emphasizing the substantial computational resources required for these methods.

**Table 7:** Training time (hours) and number of epochs required for each method.

Method	Number of epochs	Training time (h)
2D	50	2.5
Full res.	50	3.80
Low res.	50	3.20
Cascade	50	7.06
DiNTS	250	79.70
SegResNet	250	20.40
Swin UNETR	175	40.10

## Data Availability

The used dataset in this study is openly available at https://www.cancerimagingarchive.net at https://doi.org/10.7937/TCIA.e3sv-re93.

## References

[1] Chhikara B. S. Parang K., “Global Cancer Statistics 2022: the trends projection analysis,” Chem. Biol. Lett. 10(1), 451–451 (2023).

[2] Ma X. et al., “Automated fibroglandular tissue segmentation in breast MRI using generative adversarial networks,” Phys. Med. Biol. 65(10), 105006 (2020). PHMBA7 0031-9155 10.1088/1361-6560/ab7e7f 32155611

[3] Huo L. et al., “Segmentation of whole breast and fibroglandular tissue using nnU-Net in dynamic contrast enhanced MR images,” Magn. Reson. Imaging 82, 31–41 (2021). MRIMDQ 0730-725X 10.1016/j.mri.2021.06.017 34147598

[4] Gouveia P. F. et al., “Breast cancer surgery with augmented reality,” Breast 56, 14–17 (2021). 10.1016/j.breast.2021.01.004 33548617 PMC7890000

[5] Gumbs A. A. et al., “White paper: definitions of artificial intelligence and autonomous actions in clinical surgery,” Art. Int. Surg. 2(2), 93–100 (2022). 10.20517/ais.2022.10

[6] Primakov S. P. et al., “Automated detection and segmentation of non-small cell lung cancer computed tomography images,” Nat. Commun. 13(1), 3423 (2022). NCAOBW 2041-1723 10.1038/s41467-022-30841-3 35701415 PMC9198097

[r7] Rizzo S. et al., “Radiomics: the facts and the challenges of image analysis,” Eur. Radiol. Exp. 2, 36 (2018). 10.1186/s41747-018-0068-z 30426318 PMC6234198

[r8] Simantiris G. Tziritas G., “Cardiac MRI segmentation with a dilated CNN incorporating domain-specific constraints,” IEEE J. Sel. Top. Signal Process. 14(6), 1235–1243 (2020). 10.1109/JSTSP.2020.3013351

[r9] Wu B. Fang Y. Lai X., “Left ventricle automatic segmentation in cardiac MRI using a combined CNN and U-net approach,” Comput. Med. Imaging Graph. 82, 101719 (2020). 10.1016/j.compmedimag.2020.101719 32325284

[10] Dolz J. et al., “Deep CNN ensembles and suggestive annotations for infant brain MRI segmentation,” Comput. Med. Imaging Graph. 79, 101660 (2020). 10.1016/j.compmedimag.2019.101660 31785402

[11] Isensee F. et al., “nnU-Net: a self-configuring method for deep learning-based biomedical image segmentation,” Nat. Methods 18(2), 203–211 (2021).1548-7091 10.1038/s41592-020-01008-z 33288961

[12] He X. Zhao K. Chu X., “AutoML: a survey of the state-of-the-art,” Knowl.-Based Syst. 212, 106622 (2021). KNSYET 0950-7051 10.1016/j.knosys.2020.106622

[13] Ma J. Chen J., “NnUNet with region-based training and loss ensembles for brain tumor segmentation,” Lect. Notes Comput. Sci. 12962, 421–430 (2021). LNCSD9 0302-9743 10.1007/978-3-031-08999-2_36

[14] El-Hariri H. et al., “Evaluating nnU-Net for early ischemic change segmentation on non-contrast computed tomography in patients with Acute Ischemic Stroke,” Comput. Biol. Med. 141, 105033 (2022). CBMDAW 0010-4825 10.1016/j.compbiomed.2021.105033 34802712

[15] Ferrante M. et al., “Application of nnU-Net for automatic segmentation of lung lesions on CT Images and its implication for radiomic models,” J. Clin. Med. 11(24), 7334 (2022). 10.3390/jcm11247334 36555950 PMC9784875

[16] He Y. et al., “DiNTS: differentiable neural network topology search for 3D medical image segmentation,” in Proc. IEEE/CVF Conf. Comput. Vision and Pattern Recognit., pp. 5841–5850 (2021). 10.1109/CVPR46437.2021.00578

[17] Myronenko A., “3D MRI brain tumor segmentation using autoencoder regularization,” Lect. Notes Comput. Sci. 11384, 311–320 (2019). LNCSD9 0302-9743 10.1007/978-3-030-11726-9_28

[18] Hatamizadeh A. et al., “Swin UNETR: Swin transformers for semantic segmentation of brain tumors in MRI images,” Lect. Notes Comput. Sci. 12962, 272–284 (2021). LNCSD9 0302-9743 10.1007/978-3-031-08999-2_22

[19] Myronenko A. et al., “Automated head and neck tumor segmentation from 3D PET/CT HECKTOR 2022 challenge report,” Lect. Notes Comput. Sci. 13626, 31–37 (2022). LNCSD9 0302-9743 10.1007/978-3-031-27420-6_2

[20] Siddique M. M. R. et al., “Automated ischemic stroke lesion segmentation from 3D MRI,” arXiv:2209.09546 (2022).

[21] Myronenko A. et al., “Automated 3D segmentation of kidneys and tumors in MICCAI KiTS 2023 challenge,” Lect. Notes Comput. Sci. 14540, 1–7 (2023). LNCSD9 0302-9743 10.1007/978-3-031-54806-2_1

[22] Myronenko A. et al., “Aorta segmentation from 3D CT in MICCAI SEG.A. 2023 challenge,” Lect. Notes Comput. Sci. 14539, 13–18 (2023). LNCSD9 0302-9743 10.1007/978-3-031-53241-2_2

[23] Saha A. et al., “A machine learning approach to radiogenomics of breast cancer: a study of 922 subjects and 529 DCE-MRI features,” Br. J. Cancer 119(4), 508–516 (2018). BJCAAI 0007-0920 10.1038/s41416-018-0185-8 30033447 PMC6134102

[24] Zhang Y. et al., “Automatic detection and segmentation of breast cancer on MRI using mask R-CNN trained on non–fat-sat images and tested on fat-sat images,” Acad. Radiol. 29, S135–S144 (2022). 10.1016/j.acra.2020.12.001 33317911 PMC8192591

[25] Looney S. W., “A statistical technique for comparing the accuracies of several classifiers,” Pattern Recognit. Lett. 8(1), 5–9 (1988). PRLEDG 0167-8655 10.1016/0167-8655(88)90016-5

[26] Kirchhoff Y. et al., “Skeleton recall loss for connectivity conserving and resource efficient segmentation of thin tubular structures,” arXiv:2404.03010 (2024).

[27] Ouadahi F. et al., “Automatic êbroglandular tissue segmentation in breast MRI using a deep learning approach,” in Proc. of Int. Soc. of Magn. Reson. in Med. (2022).

[28] Müller-Franzes G. et al., “Fibroglandular tissue segmentation in breast MRI using vision transformers: a multi-institutional evaluation,” Sci. Rep. 13(1), 14207 (2023). SRCEC3 2045-2322 10.1038/s41598-023-41331-x 37648728 PMC10468506

[29] Hu S. et al., “Fully automated deep learning method for fibroglandular tissue segmentation in breast MRI,” (2022).

